# Future outlook of monthly maximum daily precipitation in Pakistan’s hydroclimatic zones: high-resolution insights from CMIP6 multimodel data

**DOI:** 10.1038/s41598-026-45047-6

**Published:** 2026-04-04

**Authors:** Muhammad Adnan, Firdos Khan, Muhammad Abbas, Fahad Shahzad, TianXiang Yue

**Affiliations:** 1 State Key Laboratory of Resources and Environmental Information System, Institute of Geographical Sciences and Natural Resources Research, Chinese Academy of Sciences, Beijing, 100101 China; 2 College of Resources and Environment, University of Chinese Academy of Sciences, Beijing, 101499 China; 3 School of Natural Sciences, National University of Sciences and Technology, H-12 Sector, Islamabad, 44000 Pakistan; 4 Scuola Universitaria Superiore Studi Pavia IUSS, Pavia, Italy; 5 University of Bergamo, Bergamo, Italy; 6 Precision Forestry Key Laboratory of Beijing, Beijing Forestry University, Beijing, 100083 China

**Keywords:** Extreme precipitation, CMIP6 projections, Hydroclimatic zones, Clustering, Monsoon intensification, Climate adaptation., Climate sciences, Environmental sciences, Hydrology, Natural hazards, Water resources

## Abstract

Extreme precipitation events are intensifying under climate change, driving escalating flood risks in some of the world’s most vulnerable regions. Pakistan is one of the most hydrologically diverse and flood-prone country, previous studies have largely emphasized seasonal or mean rainfall, leaving monthly maximum daily precipitation extremes (Rx1day at monthly resolution) underexplored, despite their direct role in triggering flash floods, landslides, and infrastructure failures. This study fills that gap by analyzing bias-corrected CMIP6 multi-model ensembles to project Rx1day-month precipitation extremes for SSP2-4.5 and SSP5-8.5 across seven hydroclimatic zones. Projections are assessed for the near future (2017–2044), mid-century (2045–2072), and late century (2073–2100), relative to the 1985–2014 baseline. Findings reveal strong spatial heterogeneity. Northern and northwestern highlands exhibit the largest absolute increases, with late-century monsoon monthly maxima of daily precipitation reaching approximately 130–150 mm, nearly double baseline values. Central and southern zones also experience pronounced amplification, intensifying flash-flood, riverine, and urban drainage hazards. By contrast, western arid and coastal regions show a decline in the magnitude of monthly maximum daily precipitation, punctuated by occasional high-intensity events. Intensification is most under SSP5-8.5, where both the magnitude and spatial footprint of extremes expand significantly over time. These high-resolution, zone-specific projections demonstrate that even localized shifts in extreme rainfall can compound hazard exposure, destabilize agriculture, and overwhelm water-management systems. The results provide actionable evidence for strengthening early-warning capacity, guiding resilient infrastructure planning, and informing targeted adaptation in one of the world’s most flood-exposed countries.

**Supplementary Information:**

The online version contains supplementary material available at 10.1038/s41598-026-45047-6.

## Introduction

Extreme precipitation events are among the most destructive climate-related hazards, driving devastating floods, landslides, and infrastructure damage across the globe^1–3^. Under anthropogenic climate change, the intensification of the global hydrological cycle is altering the magnitude, frequency, and spatial distribution of heavy rainfall, with pronounced impacts in monsoon-dominated and topographically complex regions^4–7^. Anthropogenic drivers such as land-use change and biomass burning, including forest fires, also contribute significantly to greenhouse gas emissions and climate variability influencing regional climate systems^8^.The Intergovernmental Panel on Climate Change (IPCC AR6) concludes with high confidence that such events will become more frequent and intense as global mean temperatures rise^9,10^. These shifts amplify flood hazards and trigger cascading socio-economic consequences, undermining agricultural productivity, water security, and disaster resilience^9,11–13^.

South Asia, home to nearly one-quarter of the global population, is a hotspot for precipitation extremes due to the combined influence of the South Asian summer monsoon, western disturbances, and complex orographic controls^14–16^. Within this region, Pakistan ranks consistently among the world’s most climate-vulnerable nations, remaining in the top tier of the Global Climate Risk Index over the past decade^8,17,18^. In addition to climate-related hazards, rapid urbanization, industrial activities, and environmental pollution further exacerbate environmental stress and vulnerability in the country^19^. Environmental pressures including deforestation, forest degradation, and land-use changes further compound ecological vulnerability in mountainous regions of the country^20^, while increasing wildfire risk under changing climate conditions^21^. More than 60% of its annual rainfall falls during the monsoon months (July-September), with marked interannual and spatial variability across its hydroclimatic zones^22,23^, (Khan and Pilz, 2018). Catastrophic floods, such as those in 2010, and 2022, displaced millions, caused multi-billion-dollar economic losses, and overwhelmed water management systems^24–27^. Since 2021, Pakistan has endured consecutive years of extreme flooding, culminating in the unprecedented 2025 events that devastated northern regions and further stressed vulnerable infrastructure^28^.

Projections from the Coupled Model Intercomparison Project Phase 6 (CMIP6) indicate that under high-emission, Pakistan’s highly diverse climatic zones, from arid deserts to glacier-fed basins, could experience substantial increases in the intensity and spatial footprint of extreme rainfall events by the end of the century^29–31^.

Several studies have investigated precipitation extremes and climate variability across Pakistan using observed records, extreme precipitation indices, and climate model projections. For example, Hussain and Lee^32^ examined regional and seasonal variability of extreme precipitation trends across Pakistan using station observations, while Bhatti et al.^33^ analyzed long-term changes in multiple ETCCDI precipitation extreme indices based on in-situ precipitation records. Regional assessments of precipitation variability and extremes have also been conducted for climatically sensitive mountainous areas, including the sub-Himalayan and Himalayan regions of Pakistan^34,35^. In addition, several studies have investigated projected changes in climate extremes using downscaled global climate models or CMIP-based frameworks^36–38^, while others have evaluated the capability of observational datasets and CMIP6 models to reproduce precipitation variability and extreme events across Pakistan^39,40^. These studies provide important insights into the behavior of precipitation extremes and climate variability across the region.

However, most national-scale assessments have emphasized mean precipitation trends or seasonal totals, often neglecting projections of daily precipitation extremes at the monthly scale, a key indicator of flash flood potential and hydrological infrastructure design^41,42^. This lack of zone-specific, scenario-based projections hampers the development of targeted, climate-resilient adaptation strategies^43,44^.

This study addresses this gap by assessing projected changes in monthly maximum daily precipitation across Pakistan’s hydroclimatic zones under SSP2-4.5 and SSP5-8.5. Using bias-corrected CMIP6 multi-model ensembles data for near, mid, and far-future periods (relative to 1985–2014), the analysis quantifies the spatial variability of extremes and identifies high-risk zones where intensified rainfall will exacerbate flood hazards and strain water-management systems. By directly linking precipitation extremes with food security, water availability, and disaster resilience, the study contributes to global adaptation priorities under the Sustainable Development Goals, particularly SDG 2 (Zero Hunger), SDG 6 (Clean Water and Sanitation), SDG 11 (Sustainable Cities and Communities), and SDG 13 (Climate Action).

## Methodology

### Study area and data sources

**Fig. 1:**
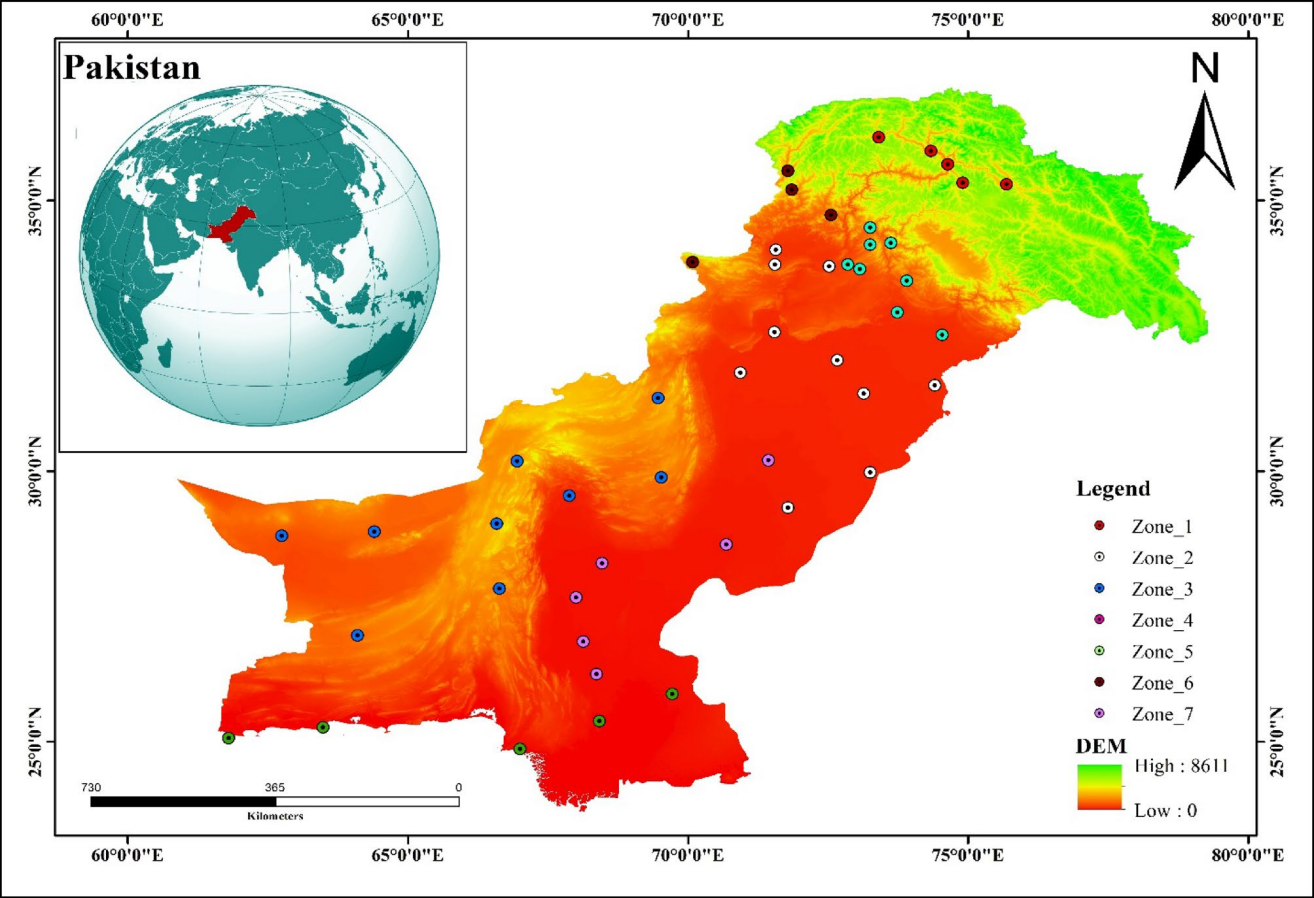
Study area map of Pakistan showing geographic extent and topography, with the meteorological stations used in the analysis. Stations are color-coded according to the seven hydroclimatic zones identified in this study.

Pakistan, located in South Asia between 23°-37°N and 60°-77°E, encompasses a diverse range of physiographic and climatic conditions. The country stretches from the high-altitude glaciated ranges to the hyper arid plateaus and coastal lowlands. The climatic variations across these regions necessitate a spatially disaggregated approach for assessment the effects of climate change on precipitation patterns. To accommodate these geographic and climatic differences, the country was divided into seven hydroclimatic zones (Table 1; Fig. 1). These zones ranging from snow-fed watersheds to arid plateaus serve as the basis for regional climate impact assessments.

**Table 1:** Climatic zone classification across Pakistan based on hierarchical clustering using geospatial and precipitation data. Each zone is characterized by distinct geographic and climatic features, with key representative meteorological stations listed.

Zone	Geographic focus	Characteristics	Key stations
1	Gilgit-Baltistan & Northern Highlands	High-altitude, glaciated, cold semi-arid to alpine	Astore, Bunji, Gilgit, Gupis, Skardu
2	Northern & Central Punjab, KP	Mid-elevation plains and foothills; semi-arid to humid subtropical	Lahore, Sargodha, Peshawar, Bahawalpur, DI Khan, Bahawalnagar, Cherat, Mianwali, Faisalabad, Kohat
3	Western Balochistan	Arid highlands and deserts; extremely low rainfall	Quetta, Kalat, Dalbandin, Khuzdar, Barkhan, Sibbi, Zhob, Panjgur, Nokkandi
4	Potohar, Northern Punjab & Kashmir	Mid-elevation humid valleys and plains	Islamabad, Murree, Jehlum, Balakot, Kotli, Kakul, Ghari Dupatta, Sialkot
5	Southern Coast (Sindh & Makran)	Coastal lowlands; arid and maritime influence	Karachi, Hyderabad, Pasni, Jiwani, Chhor
6	Northwestern Highlands (Dir, Swat)	Mountainous, snow-fed basins; high rainfall	Dir, Saidu Sharif, Darosh, Parachinar
7	Southern Punjab & Upper Sindh	Hot arid plains, agriculturally important, monsoon-influenced	Multan, Jacobabad, Rohri, Khanpur, Nawabshah, Padidan

Daily precipitation data from 1985 to 2015 were collected from the Pakistan Meteorological Department (PMD), spanning 47 meteorological stations across the country. These historical datasets were critical for validating model outputs and capturing climate variability. Daily precipitation was used to derive monthly maximum daily precipitation by extracting, for each calendar month and year, the single largest daily precipitation value, equivalent to the ETCCDI Rx1day index evaluated at monthly resolution. Additionally, GCM outputs were obtained from the Coupled Model Intercomparison Project Phase 6 (CMIP6), covering two Shared Socioeconomic Pathways (SSPs): SSP2-4.5 and SSP5-8.5. The projections were analyzed for three time periods: near-future (F1: 2017–2044), mid-future (F2: 2045–2072), and far-future (F3: 2073–2100). Seven CMIP6 models, BCC-CSM2-MR, CMCC-ESM2, GFDL-CM4, GFDL-ESM4, INM-CM4-8, MRI-ESM2-0, NESM3, were selected based on their historical performance in replicating Pakistan’s climatic conditions and availability of the data for the entire considered duration. Key characteristics of the selected CMIP6 models, including modeling centers and nominal atmospheric resolution, are summarized in Table 2. Outputs from these models were synthesized into a robust multi-model ensemble using K-fold cross-validation to improve the reliability of future projections.

**Table 2:** List of CMIP6 global climate models (GCMs) used in this study, including the modeling center and nominal horizontal atmospheric resolution. All models provide daily precipitation outputs for historical and future simulations under SSP2-4.5 and SSP5-8.5 and were selected based on data availability and demonstrated performance in representing Pakistan’s regional climate.

No.	Model name	Modeling center / institution	Country	Nominal atmospheric resolution
1.	BCC-CSM2-MR	Beijing Climate Center (BCC)	China	~1.1° × 1.1°
2.	CMCC-ESM2	Centro Euro-Mediterraneo sui Cambiamenti Climatici (CMCC)	Italy	~1.0° × 1.0°
3.	GFDL-CM4	NOAA Geophysical Fluid Dynamics Laboratory	USA	~1.0° × 1.0°
4.	GFDL-ESM4	NOAA Geophysical Fluid Dynamics Laboratory	USA	~1.0° × 1.0°
5.	INM-CM4-8	Institute of Numerical Mathematics (INM)	Russia	~2.0° × 1.5°
6.	MRI-ESM2-0	Meteorological Research Institute (MRI)	Japan	~1.1° × 1.1°
7.	NESM3	Nanjing University of Information Science & Technology	China	~1.9° × 1.9°

### Methodological framework

**Fig. 2:**
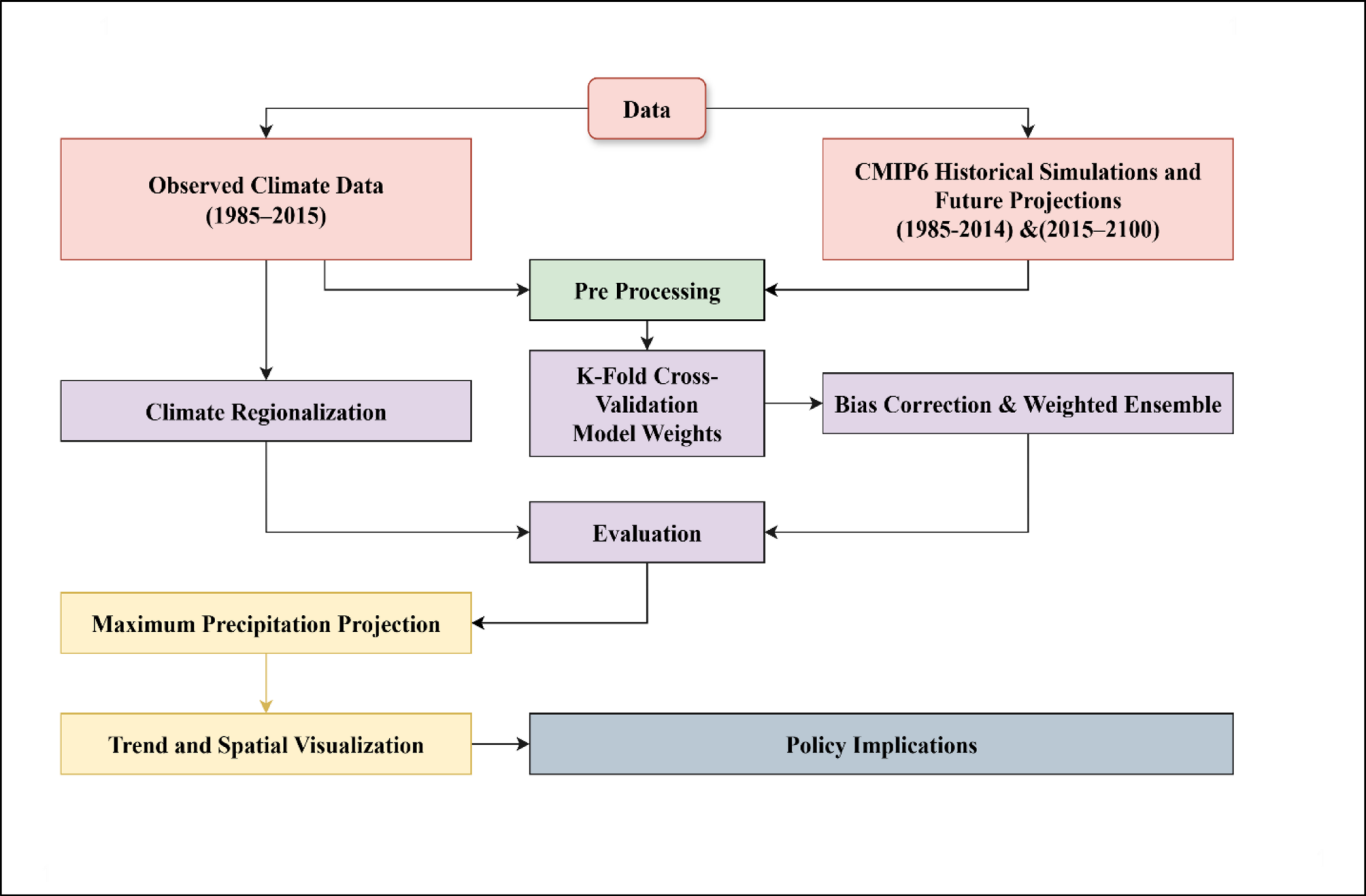
Schematic overview of the methodological framework employed for regional precipitation analysis under climate change scenarios across Pakistan.

A schematic overview of the analytical workflow is presented in Fig. 2, outlining the sequential integration of observed data, climate model projections, regionalization, ensemble evaluation, and precipitation trend diagnostics. This flowchart encapsulates the study’s systematic approach to assessing projected precipitation characteristics across Pakistan under changing climate conditions.

### Data preprocessing and climate regionalization

**Fig. 3:**
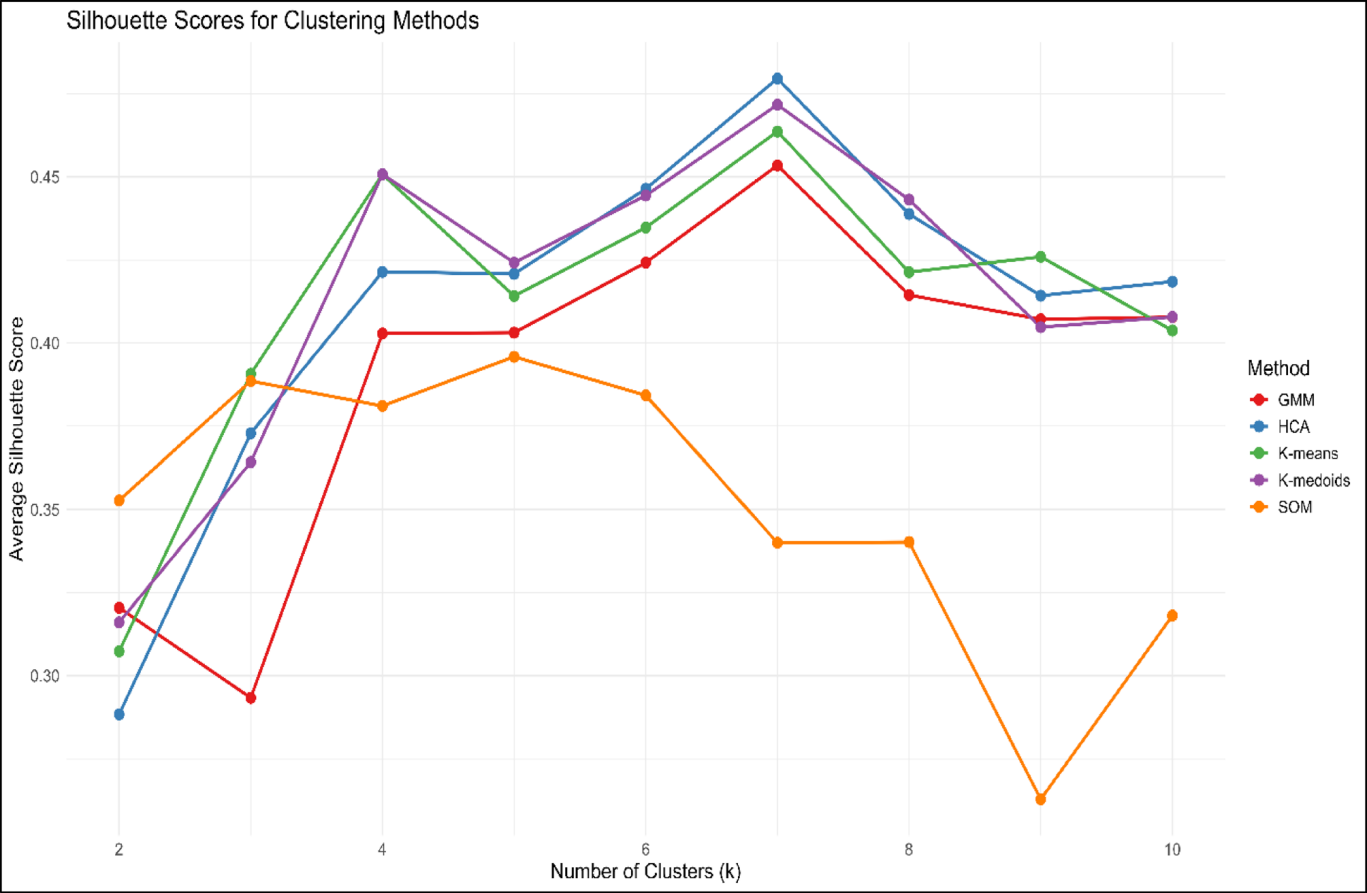
Hydroclimatic zones of Pakistan derived from hierarchical clustering based on latitude, longitude, elevation, and mean annual precipitation.

Before analyzing the data, rigorous preprocessing was conducted to ensure its accuracy. Anomalies and missing values ​​in the raw datasets were identified and corrected using standard quality control procedures. Station-level quality control was applied prior to analysis to ensure the reliability of precipitation extremes. Stations with more than 10% missing daily records over the reference period were excluded. For remaining stations, short gaps (≤ 3 consecutive days) were infilled using neighboring-station ratios, while longer gaps were left missing. Outliers were identified using percentile-based thresholds and manual consistency checks against nearby stations. To preserve the integrity of extremes, no infilling or adjustment was applied to days contributing to monthly maximum daily precipitation (Rx1day-month). No formal homogenization correction was applied, as the analysis focuses on extreme values rather than long-term means, and all detected inhomogeneities were handled conservatively through quality control rather than statistical adjustment. The climate regionalization of Pakistan was carried out using unsupervised clustering techniques. We evaluated several clustering algorithms, including K-Means, K-Medoids, Gaussian Mixture Models (GMM), Self-Organizing Maps (SOM), and Hierarchical Cluster Analysis (HCA). Based on the quality of the clusters as assessed by the silhouette coefficient, HCA was selected as the most effective method for capturing the country’s climatic heterogeneity. The key features used for clustering were latitude, longitude, elevation, and mean annual precipitation. The clustering results were validated using the elbow method, and the optimal number of clusters was determined by examining the inter-cluster distances and silhouette scores (Fig. 3). The resulting seven hydroclimatic zones are detailed in Table 1, each representing a distinct geographic and climatic area. These zones serve as the basis for regional climate analysis and enable the identification of location-specific precipitation trends, which are critical for assessing the regional impacts of climate change.

### Bias correction of CMIP6 precipitation data

Statistical bias correction was applied to daily CMIP6 precipitation outputs using the Quantile Delta Mapping (QDM) method, which corrects systematic biases while preserving projected climate change signals in precipitation extremes. QDM adjusts modeled precipitation distributions by mapping quantiles of simulated historical data to observations and subsequently applying the modeled relative change to future projections^45^.

Bias correction was performed at the station scale, where daily precipitation outputs from each CMIP6 model were extracted only at locations corresponding to the 47 Pakistan Meteorological Department (PMD) stations used in this study. For each station, precipitation time series were obtained from the nearest model grid cell based on geographic proximity, ensuring a direct one-to-one correspondence between observed and modeled data. Station–grid matching was performed prior to bias correction. Correction functions were derived over the historical reference period 1985–2014 using observed daily precipitation and corresponding model-simulated historical data, and were subsequently applied to future projections under SSP2-4.5 and SSP5-8.5, assuming bias stationarity.

Bias correction was applied only to precipitation, and the resulting bias-corrected daily time series were subsequently used to compute monthly maximum daily precipitation (Rx1day-month) for all analyses.

### Ensemble construction and model evaluation

To enhance the robustness of climate projections, we employed a weighted ensemble approach that integrates outputs from seven selected GCMs. In this framework, each model output, denoted as *Ni*${(\mathrm{N}}_{1,}{\mathrm{N}}_{2}{\dots\dots\mathrm{N}}_{8})$ assigned a corresponding non-negative weight ​${{\mathrm{w}}_{\mathrm{i}}(\mathrm{w}}_{1,}{\mathrm{w}}_{2}{\dots\dots\mathrm{w}}_{\mathrm{k}}$,) based on its predictive performance. The weights represent normalized skill-based coefficients derived from regression performance and were constrained to be non-negative and to sum to unity. High-skill models therefore exert greater influence on the final projection, while models with limited accuracy contribute minimally. The weighted ensemble mean was calculated using the standard formulation given in Eq. 1.

1 $$\mathrm{X}=\frac{{\sum}_{\mathrm{i}=1}^{8}{\mathrm{w}}_{\mathrm{i}}{\mathrm{N}}_{\mathrm{i}}}{{\sum}_{\mathrm{i}=1}^{8}{\mathrm{w}}_{\mathrm{i}}}$$

Here, $\mathrm{X}$ represents the final weighted ensemble projection ${\mathrm{N}}_{\mathrm{i}}$ ​the output of the i-th GCM and ${\mathrm{w}}_{\mathrm{i}}$ ​its assigned weight. Negative regression coefficients were not permitted to contribute to the ensemble; models associated with negative or negligible skill were assigned zero or near-zero weights after normalization to maintain physical consistency.

To systematically determine these weights, we applied K-fold cross-validation (KFCV) in conjunction with a regression-based evaluation framework. Bias-corrected daily precipitation from each GCM, extracted at station locations, was used as predictor variables, while observed daily station precipitation served as the target variable during the historical period. In each iteration, the GCM outputs served as predictors in a multivariate linear regression model given in Eq. 2.

2 $$\mathrm{y}=\mathrm{X}{\uptheta}+{\upepsilon}$$

In this equation, $\mathrm{y}$ represents the vector of observed values, *X* is the matrix of predictor variables, ${\uptheta}=({\mathrm{X}}^{\mathrm{T}}\mathrm{X}{)}^{-1}{\mathrm{X}}^{\mathrm{T}}\mathrm{y}$ denotes the regression coefficients, and ${\upepsilon}$ is the error term. Regression coefficients were used only to infer relative model skill for weighting purposes, acknowledging potential collinearity among GCM predictors that simulate the same physical processes. Model performance was quantified using the Root Mean Square Error (RMSE), with lower RMSE values ​​indicating higher predictive skill.

**Fig. 4:**
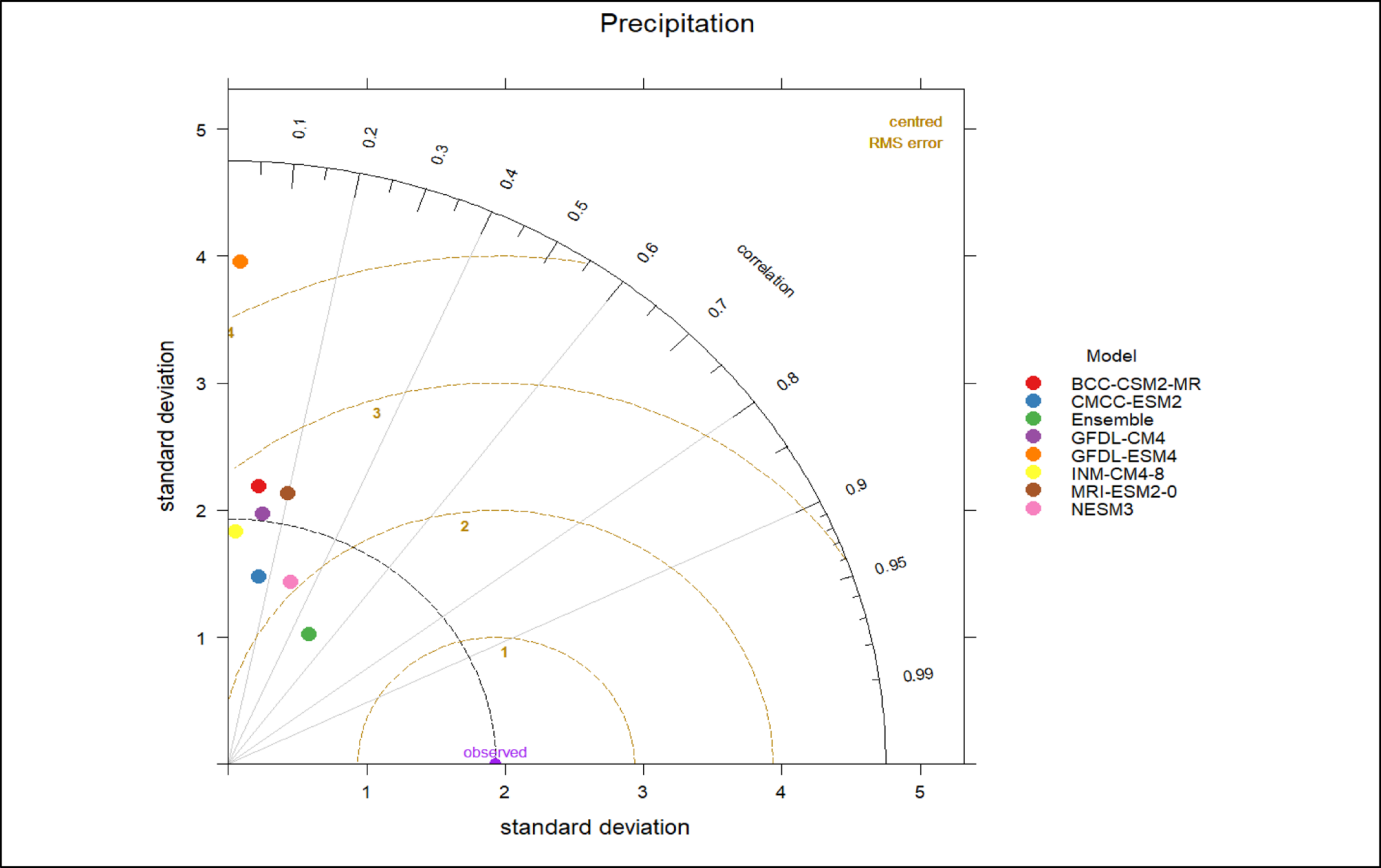
Taylor diagram evaluating the performance of individual CMIP6 models and the weighted ensemble mean against observed monthly maximum daily precipitation (Rx1day at monthly resolution) for the historical period 1985–2014, based on correlation coefficient, standard deviation, and root mean square error, using monthly samples (30 years × 12 months).

Finally, the reliability of both the ensemble and individual GCMs was evaluated using Taylor diagrams (Fig. 4), which provide a concise comparison of correlation coefficients, RMSE, and standard deviations relative to observations. The ensemble consistently outperformed individual models, confirming its suitability for capturing precipitation extremes across Pakistan’s diverse hydroclimatic zones.

This weighting strategy prioritizes models with higher skill in reproducing observed precipitation variability, thereby improving the robustness of ensemble estimates of precipitation extremes across Pakistan’s diverse hydroclimatic zones. Inter-model variability is reported alongside ensemble means using the range of individual GCM projections to provide a measure of projection uncertainty.

### Trend analysis

To assess long-term changes in rainfall behavior time series of monthly daily precipitation maxima were derived from the historical (1985–2014) and projected (SSP2-4.5 and SSP5-8.5) datasets for each hydroclimatic zone. Two widely used non-parametric statistical techniques were applied: Kendall’s Tau to detect monotonic trends in precipitation series and Sen’s Slope estimator to quantify the magnitude of change over time. These methods were selected for their robustness to non-normal data distributions and outlier sensitivity, ensuring reliable detection of directional shifts in precipitation extremes.

Spatial visualization of trend characteristics was performed using the Inverse Distance Weighting (IDW) interpolation technique, which generates continuous gridded surfaces from station-level estimates of Sen’s slope, Kendall’s Tau, and their corresponding p -values. A power parameter of *p* = 2 was employed, balancing smoothness and local representativeness of interpolated values. The resulting interpolated layers provided spatially explicit maps of trends in monthly maximum daily precipitation across Pakistan, highlighting zones of intensification, weakening of extreme precipitation intensity, and increased variability. Statistical significance from the Mann–Kendall test was visualized using categorized p-value levels to represent different confidence levels in detected trends. Such representations are commonly used in hydro-climatic trend analyses to highlight both statistically significant and emerging spatial signals^46–48^.

These visualizations, combined with trend diagnostics, offer a coherent basis for interpreting the spatiotemporal evolution of precipitation extremes. Together, they facilitate identification of regional hotspots where projected increases in rainfall maxima are most likely to amplify hydrological hazards such as flash flooding, landslides, and urban drainage overload, as well as regions where trends indicate weakening or increased variability in extreme precipitation intensity.

## Results

### Projected monthly maximum daily precipitation patterns across Pakistan

**Fig. 5:**
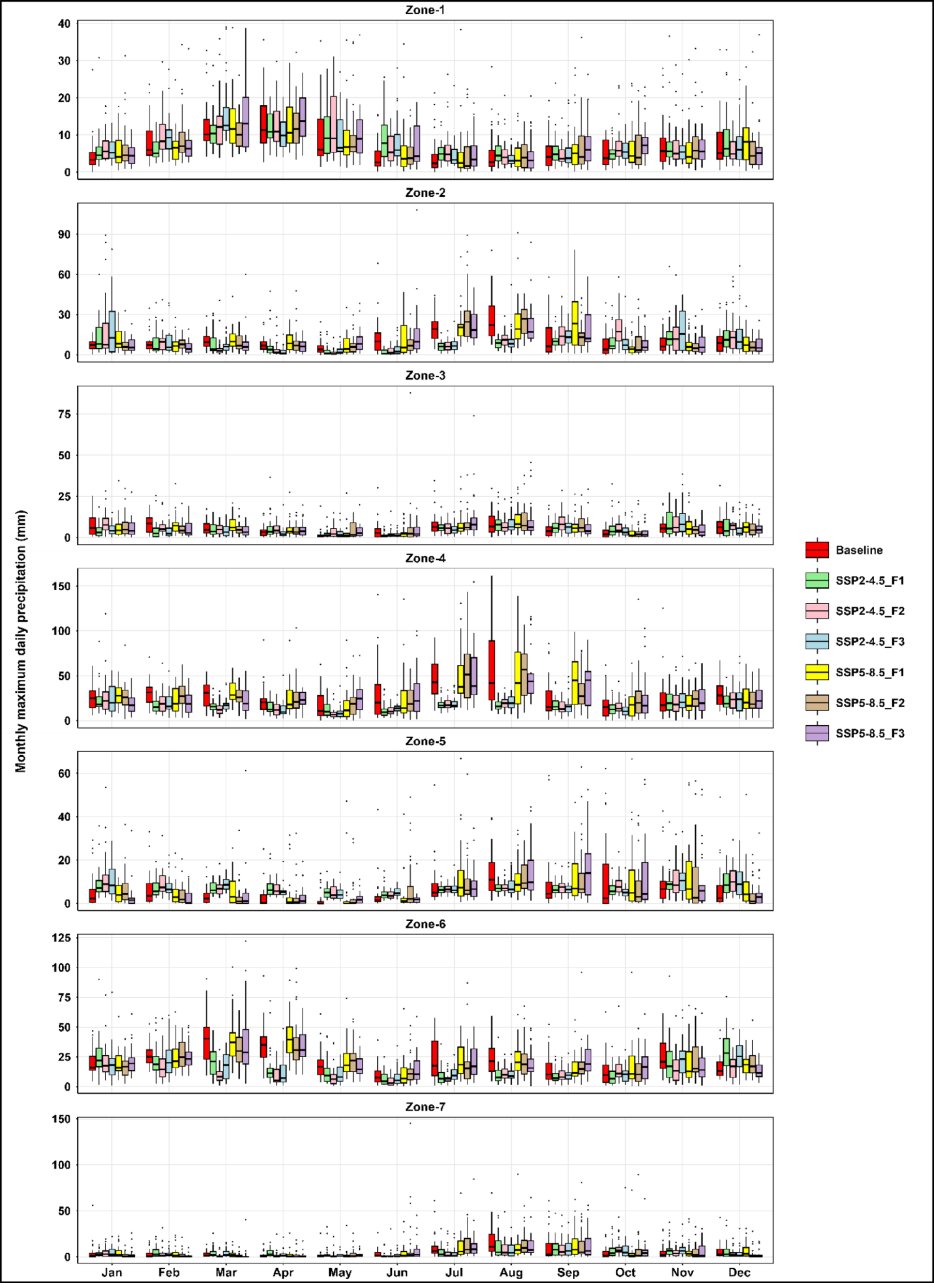
Monthly maximum daily precipitation (Rx1day at monthly resolution, mm) across Pakistan’s seven hydroclimatic zones for the baseline period (1985–2014) and future climate scenarios (SSP2-4.5 and SSP5-8.5) during the near-future (F1: 2017–2044), mid-century (F2: 2045–2072), and late-century (F3: 2073–2100) periods.

Projected monthly maxima of daily precipitation exhibits marked spatial heterogeneity and an increase in the magnitude and variability of extremes under future climate scenarios (Fig. 5), most notably under SSP5-8.5 by the late century (F3: 2073–2100). This intensification is concentrated during the core monsoon season (July-September), when rainfall-driven hazards are historically highest and recent event confirm this.

Zones 4 (Potohar, Northern Punjab, Kashmir) and 7 (Southern Punjab, Upper Sindh) exhibit the largest projected increases in magnitude, with projected maxima of ~ 130–150 mm in July-September, nearly double the baseline (60–80 mm; Fig. 5). In the steep, landslide-prone Zone 6 (Northwestern Highlands), monsoon peaks reach ~ 110–125 mm, heightening slope-failure and runoff risks. Zone 2 (Central/Northern Punjab, KP) shows moderate gains (70–90 mm) and wider variability. Zones 3 (Western Balochistan) and 5 (Southern Coast) remain comparatively dry but experience episodic maxima of 60–75 mm and 50–60 mm, particularly in late monsoon months. Zone 1 (Gilgit-Baltistan, Northern Highlands) remains largely stable, though minor increases (30–40 mm) may influence glacier melt and downstream flows.

Across all zones, projections indicate broadened interquartile ranges and more frequent outliers (Fig. 5), especially under SSP5-8.5, reflecting rising rainfall volatility and year-to-year unpredictability compared to the baseline.

### Historical trends in monthly maximum daily precipitation (1985–2014)

**Fig. 6:**
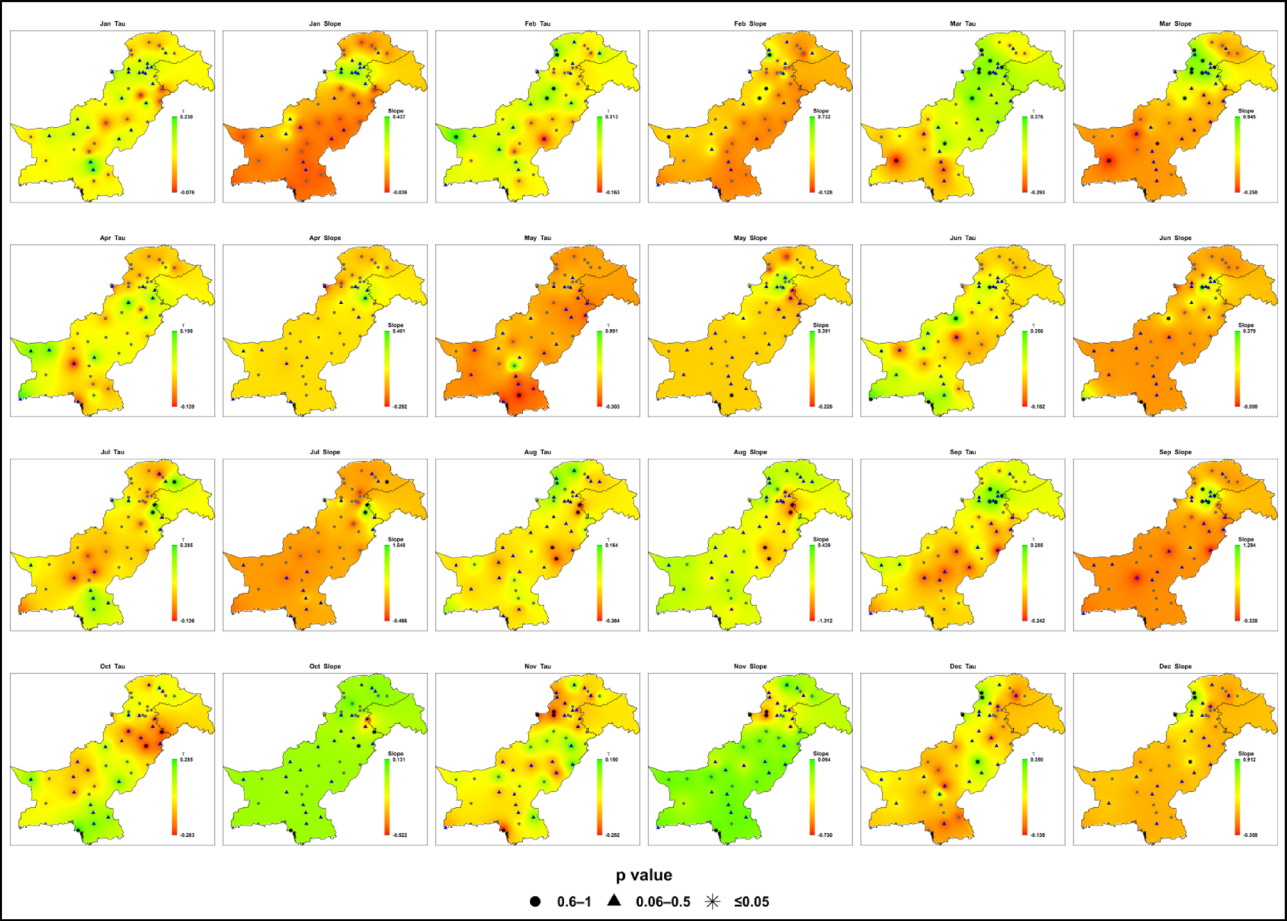
Spatial distributions of Kendall’s Tau (trend direction and strength) and Sen’s slope (mm month⁻¹) for monthly maximum daily precipitation during the baseline period (1985–2014) across Pakistan. Kendall’s Tau and Sen’s slope are spatially interpolated, while p-values are displayed at station locations using symbols to indicate statistical significance (★ *p* ≤ 0.05; ▲ 0.06–0.5; ● 0.6–1).

Analysis of monthly maximum daily precipitation during the baseline period (1985–2014) reveals weak and statistically insignificant trends across all seven hydroclimatic zones given in Fig. 6; Table S1. Sen’s slope values ​​range from − 1.30 to + 1.57 mm/month, with Kendall’s Tau between − 0.36 and + 0.37. For most months and regions, P-values exceeding 0.05 indicate that precipitation variability is largely stationary across most months and regions.

During the monsoon season (July-September), only isolated upward trends are observed. Zone 4 (Potohar, northern Punjab, Kashmir) shows a July peak of + 1.57 mm/month (Tau = + 0.23, *P* ≈ 0.03), while Zone 7 (southern Punjab, upper Sindh) records a September increase of + 1.3 mm/month. Zone 6 (northwestern highlands) shows moderate increases in August-September (up to + 0.47 mm/month), but July often indicates a localized decline in monthly maximum daily precipitation (as low as − 0.45 mm/month).

In the pre-monsoon months (April-June), variability is minor: Zone 2 (central Punjab, KP) records scattered positive slopes, most notably in February (+ 0.73 mm/month), while Zones 3 (western Balochistan) and 5 (southern coast) show weak negative trends in monthly maximum daily precipitation (-0.22 to -0.28 mm/month). Winter (January-February) and autumn (October-November) display stable or statistically insignificant changes, including at coastal stations such as Karachi and Pasni (Zone 5, slopes down to -0.34 mm/month).

Zone 1 (Gilgit-Baltistan, northern highlands) remains stable, with all slopes between − 0.04 and + 0.44 mm/month. Figure 6 confirms that no consistent upward or downward trend is detectable during the baseline, providing a neutral reference against which future projections can be evaluated.

### Projected trends analysis under SSP2-4.5

#### Near-future (2017–2044)

**Fig. 7:**
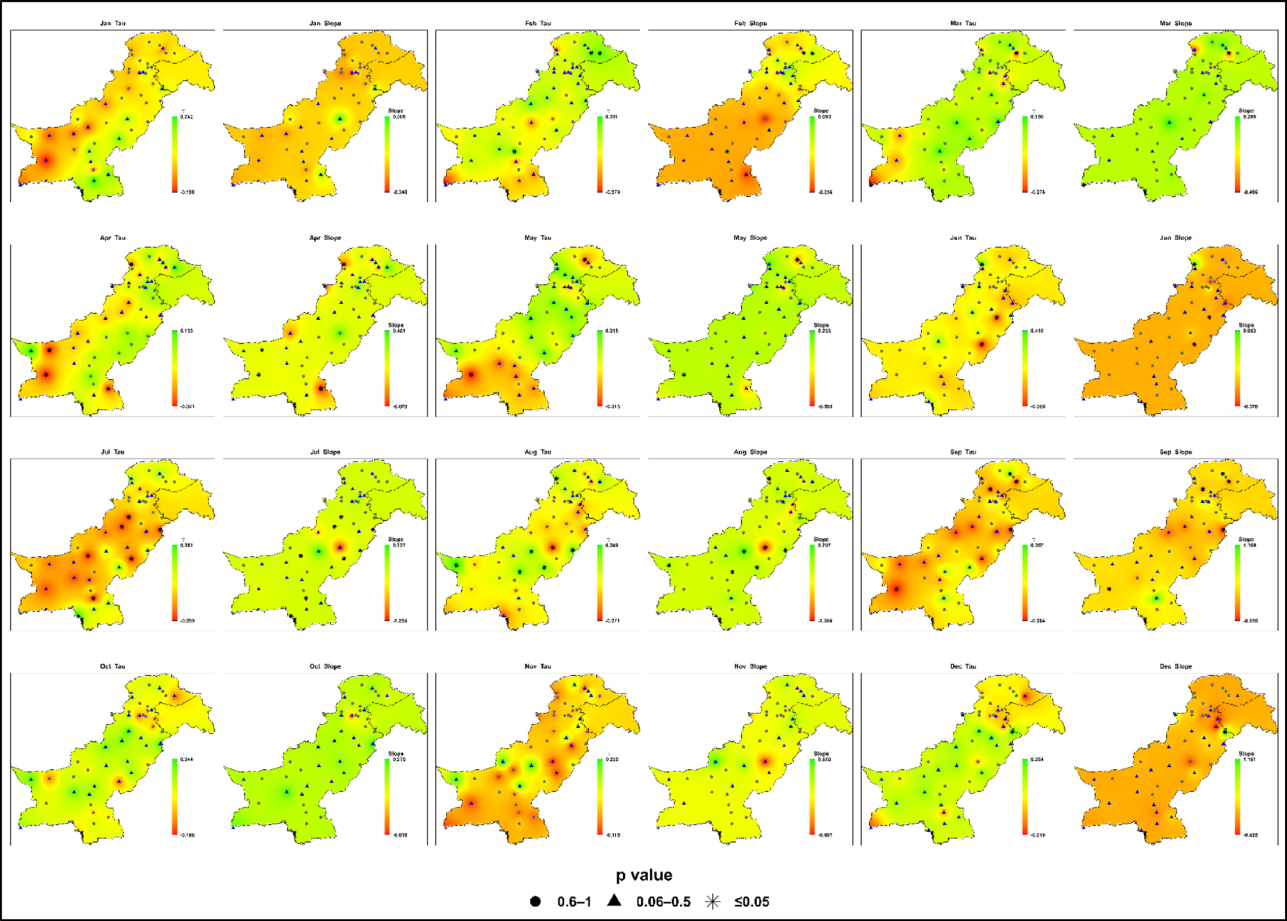
Spatial distributions of Kendall’s Tau and Sen’s slope (mm month⁻¹) for monthly maximum daily precipitation across Pakistan under SSP2-4.5 during the near-future period (2017–2044). Kendall’s Tau and Sen’s slope are interpolated; statistical significance is indicated by station-based p-value symbols ((★ *p* ≤ 0.05; ▲ 0.06–0.5; ● 0.6–1).

Projections for the near-future (2017–2044) under SSP2-4.5 indicate weak and statistically insignificant changes in monthly maximum daily precipitation across most of Pakistan in Fig. 7; Table S2. Sen’s slope values ​​range from − 1.27 to + 1.18 mm/month, with Kendall’s Tau between − 0.30 and + 0.30, confirming limited evidence of emerging trends.

During the monsoon season (July-September), only localized signals appear. Zone 4 (Islamabad-Murree-Jhelum) records modest increases, while Zone 7 (southern Punjab-upper Sindh) shows delayed intensification, with July and September slopes occasionally exceeding + 1.0 mm/month. Zone 6 (northwestern highlands) exhibits small July gains (+ 0.4 mm/month) but localized negative trends in monthly maximum daily precipitation in August (down to − 1.26 mm/month).

In non-monsoon months, weak or negative tendencies dominate. Zones 3 and 5 (western Balochistan and southern coast) show mild declines in monthly maximum daily precipitation during spring (March-May) and October (-0.3 to -0.6 mm/month). Zone 2 (central Punjab, KP) displays scattered minor increases in February and May without spatial consistency, while Zone 1 (high-altitude north) remains largely stable (-0.1 to + 0.3 mm/month).

The results presented in Fig. 5 confirm that interannual spread and extreme outliers remain comparable to the baseline, indicating limited changes in rainfall volatility. Localized monsoon intensification signals are emerging in northeastern and southern regions, indicating early hydroclimatic contrasts, though no coherent regional pattern has yet developed.

#### Mid-century (2045–2072)

**Fig. 8:**
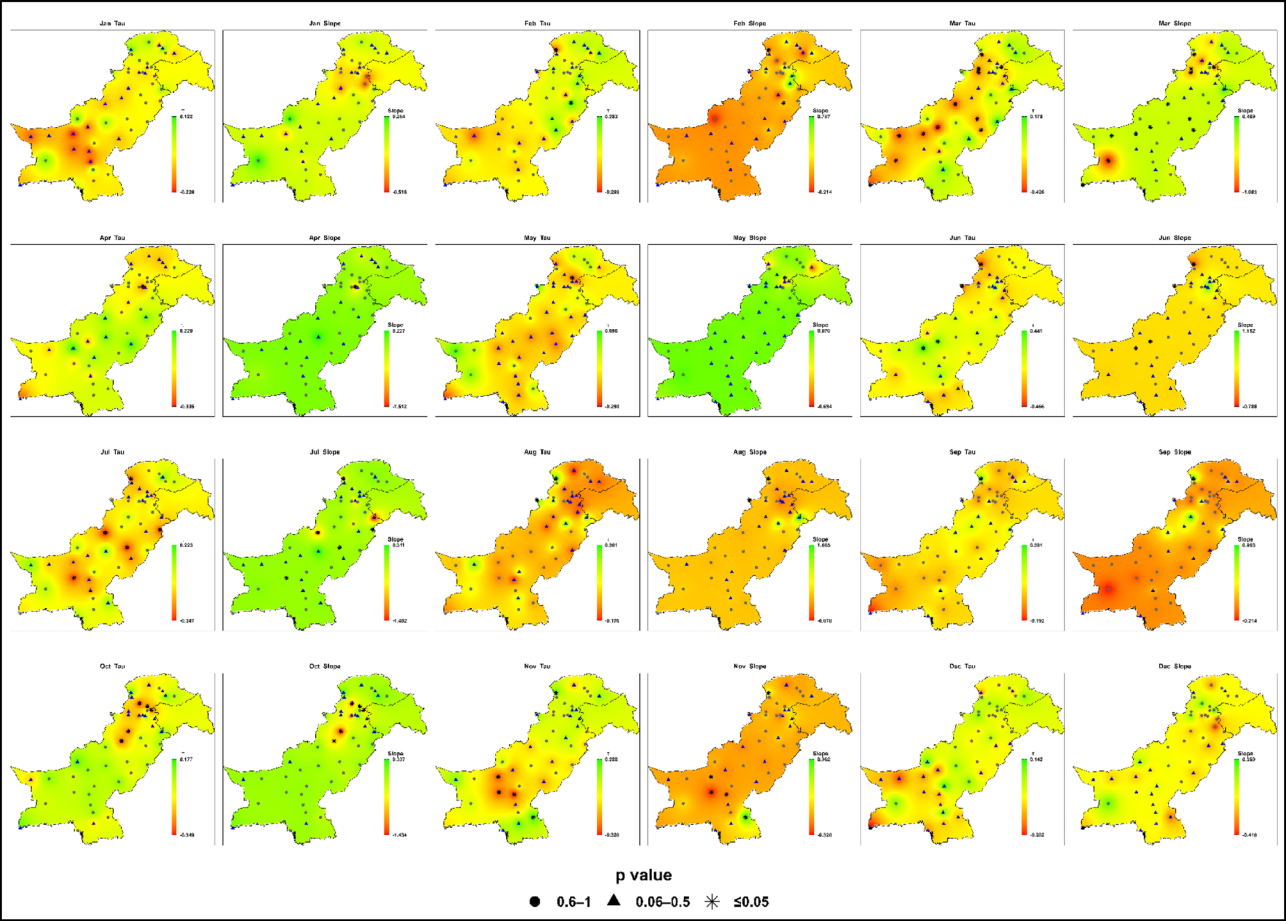
Spatial distributions of Kendall’s Tau and Sen’s slope (mm month⁻¹) for monthly maximum daily precipitation across Pakistan under SSP2-4.5 during the mid-century period (2045–2072). P-value symbols denote station-level statistical significance (★ *p* ≤ 0.05; ▲ 0.06–0.5; ● 0.6–1).

Projected trends for 2045–2072 under SSP2-4.5 reveal greater spatial and seasonal variability in monthly maximum daily precipitation given in Fig. 8 and Table S3. Sen’s slope values ​​span − 1.58 to + 1.67 mm/month, with Kendall’s Tau from − 0.48 to + 0.44. While the majority of trends remain statistically insignificant (*P* > 0.05), localized regional signals begin to emerge, despite limited statistical significance at the grid scale.

During the monsoon (July-September), intensification is most prominent in Zone 4 (central/northeastern Pakistan), where August exhibits the strongest increase (+ 1.67 mm/month, Tau = + 0.31, *P* ≈ 0.02). Zone 7 (southern Punjab-upper Sindh) also registers notable gains in August (+ 0.99 mm/month, Tau ≈ + 0.40, *P* = 0.008), exceeding baseline values. In contrast, southern Zone 4 and Zone 6 (northwestern highlands) record localized declines in monthly maximum daily precipitation in July (down to − 1.41 mm/month), underscoring pronounced spatial inconsistency.

In the pre-monsoon season (April–June), Zone 3 (western Balochistan) shows a weakening of extreme precipitation intensity, with April slopes reaching − 1.58 mm/month near Quetta and Kalat. Zone 5 (southern coast) also presents persistent April–June declines (− 0.69 to − 1.46 mm/month), indicating reduced intensity of extreme rainfall events during this period. Other regions, including high-altitude Zone 1, remain comparatively stable (-0.21 to + 0.49 mm/month). Winter and autumn months show negligible or insignificant changes across all zones.

Boxplot analysis for mid-century highlights widened interquartile ranges and more frequent outliers during the monsoon, signaling the onset of rainfall variability and greater extreme-event risk in key regions.

By mid-century, Pakistan faces localized increases in monsoon-related precipitation extremes, accompanied by increasing variability in the northeast and south, persistent weakening of extreme precipitation intensity along western and coastal margins, and continued stability elsewhere. While most grid-point trends remain statistically insignificant, the growing variability marks the early stages of a shifting hydroclimate.

#### Late-century (2073–2100)

**Fig. 9:**
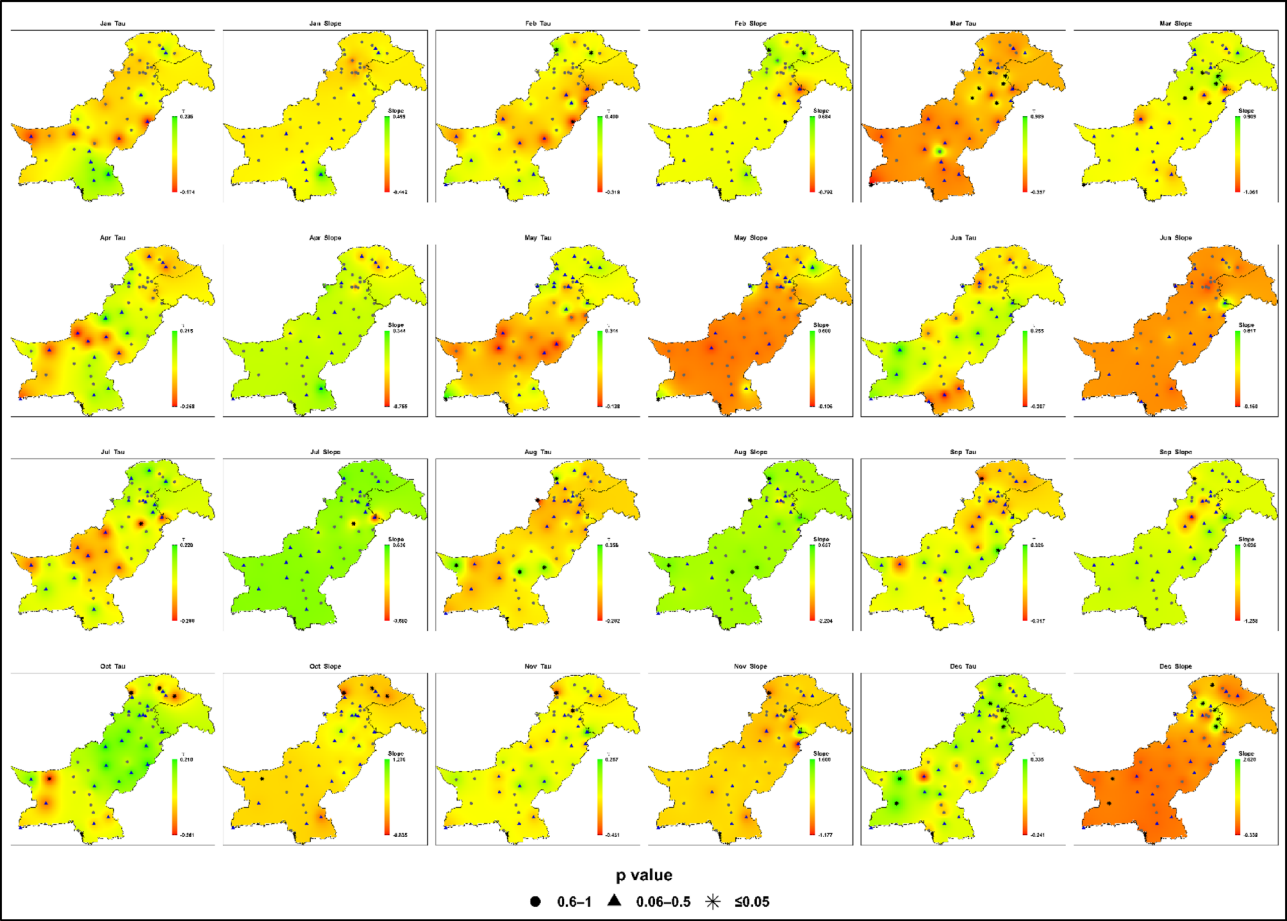
Spatial distributions of Kendall’s Tau and Sen’s slope (mm month⁻¹) for monthly maximum daily precipitation (Rx1day at monthly resolution) across Pakistan under SSP2-4.5 during the late-century period (2073–2100). Statistical significance is represented by p-value symbols (★ *p* ≤ 0.05; ▲ 0.06–0.5; ● 0.6–1) at station locations.

Late-century projections (2073–2100) under SSP2-4.5 reveal pronounced spatial heterogeneity and clear intensification of extreme precipitation across Pakistan’s hydroclimatic zones (Fig. 9; Table S4). Sen’s slopes range from − 2.33 to + 1.46 mm/month and Kendall’s Tau from − 0.44 to + 0.35, though statistically significant trends remain uncommon at the grid scale.

During the monsoon season (July-September), intensification persists and strengthens in Zone 4 (central/northeastern Pakistan) and Zone 7 (southern Punjab-upper Sindh), where maxima frequently reach 130–150 mm, nearly double the baseline in some areas. Zone 6 (northwestern highlands) also records substantial increases in July-August (110–125 mm), amplifying flash-flood and landslide risks. Moderate gains are projected for Zone 2 (central/northern Punjab, KP; maxima 70–90 mm), with broader spread and more outliers compared to the baseline.

Zones 3 (western Balochistan) and 5 (southern coast) remain comparatively low in average monthly maximum daily precipitation but exhibit isolated high-intensity rainfall events of approximately 60–75 mm and 50–60 mm, respectively, predominantly during August–September. By contrast, the high-altitude Zone 1 (Gilgit-Baltistan, northern highlands) remains comparatively stable (30–40 mm), with only slight increases that could still influence glacier melt and downstream hydrology.

Outside the monsoon months, including winter and autumn, most regions display neutral or weak trends. However, late-century boxplots show sharply widened interquartile ranges and more frequent extreme outliers during the monsoon, underscoring rising rainfall variability.

### Projected trends analysis under SSP5-8.5

#### Near-future (2017–2044)

**Fig. 10:**
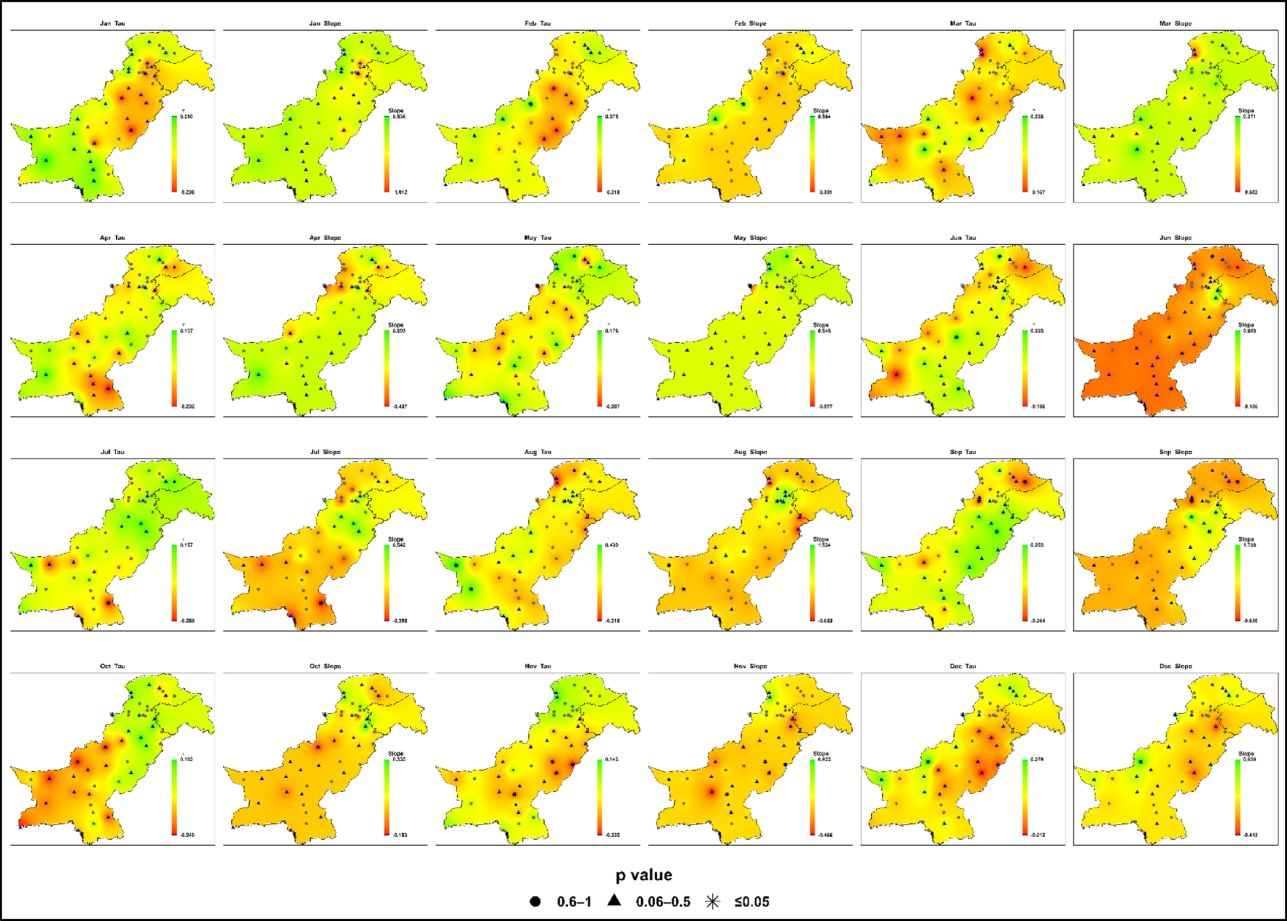
Spatial distributions of Kendall’s Tau and Sen’s slope (mm month⁻¹) for monthly maximum daily precipitation (Rx1day at monthly resolution) across Pakistan under SSP5-8.5 during the near-future period (2017–2044). Station-level p-value symbols indicate statistical significance ((★ *p* ≤ 0.05; ▲ 0.06–0.5; ● 0.6–1).

Projections for the near-future period (2017–2044) under SSP5-8.5 indicate generally weak and statistically insignificant changes in monthly maximum daily precipitation across Pakistan (Fig. 10; Table S5). However, early signals of intensification are emerging.

During the core monsoon season (July-September), localized increases are evident in Zone 4 (Potohar, northern Punjab, Kashmir) and Zone 7 (southern Punjab, upper Sindh), where maxima approach 100–120 mm, exceeding baseline values ​​and marking the initial onset of monsoon amplification. Zone 6 (northwestern highlands) shows modest July gains (up to + 0.5 mm/month), while August and September reveal patchy negative trends in monthly maximum daily precipitation and spatial variability.

Outside the monsoon, Zones 3 (western Balochistan) and 5 (southern coast) exhibit weak-to-moderate declines in monthly maximum daily precipitation during spring, with declines reaching − 0.88 mm/month in some locations. Zone 2 (central Punjab, KP) registers only isolated, minor increases (notably in February), and Zone 1 (high-altitude north) remains largely stationary. Across non-monsoon months, changes are minimal or absent.

The results given in Fig. 5 confirm that interannual spread and the frequency of extreme outliers remain similar to the baseline, suggesting that widespread shifts in rainfall volatility have not yet materialized. Nonetheless, the observed variability in monsoon-prone areas signals the early stages of a transition toward a more volatile hydroclimate.

Thus, near-future SSP5-8.5 projections suggest broad stability, the localized intensification in the northeast and south provides an early warning of emerging hydroclimatic contrasts, even if statistical significance is not yet widespread.

#### Mid-century (2045–2072)

**Fig. 11:**
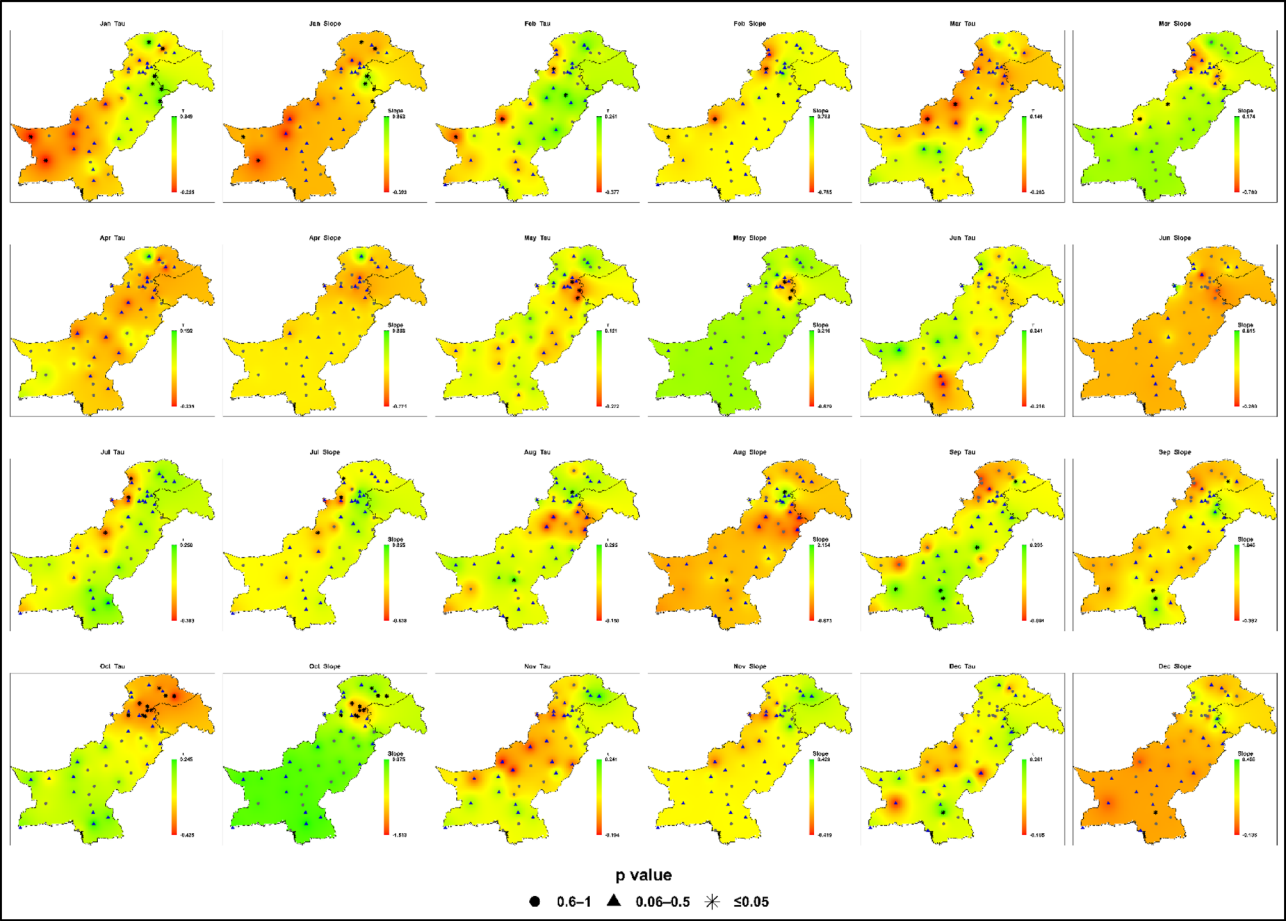
Spatial distributions of Kendall’s Tau and Sen’s slope (mm month⁻¹) for monthly maximum daily precipitation (Rx1day at monthly resolution) across Pakistan under SSP5-8.5 during the mid-century period (2045–2072). Statistical significance is shown using p-value symbols (★ *p* ≤ 0.05; ▲ 0.06–0.5; ● 0.6–1) at station locations.

By mid-century, projections under SSP5-8.5 presented Fig. 11 and Table S6 reveal clearer spatial and seasonal contrasts in monthly maximum daily precipitation, though most trends remain statistically insignificant at the grid scale.

Monsoon intensification is strongest in Zone 4 (Potohar, northern Punjab, Kashmir) and Zone 7 (southern Punjab, upper Sindh), where July-August maxima frequently exceed 120–140 mm, and local slopes reach up to + 2.16 mm/month, nearly double the baseline in key hotspots. Zone 6 (northwestern highlands) also experiences pronounced extremes, with peaks approaching 120 mm, though spatial patterns remain variable and localized negative trends in monthly maximum daily precipitation persist in some areas.

Spring and early summer show strengthened declines in monthly maximum daily precipitation in Zones 3 (western Balochistan) and 5 (southern coast), where slopes fall below − 1.0 mm/month, although rare convective outliers (up to 75 mm) still occur. Zone 2 (central Punjab, KP) records moderate monsoon increases (70–90 mm) and rising event-to-event variability, while Zone 1 (high-altitude north) remains comparatively stable with only minor gains.

The results given in Fig. 5 for F2 show broadened interquartile ranges and more frequent outliers during the monsoon indicating the onset of a more unstable rainfall regime in the most exposed zones.

In summary, mid-century SSP5-8.5 projections highlight expanding monsoon extremes and growing interannual volatility in central/northeastern and southern Pakistan, reduced intensity of extreme rainfall events in the arid west and coastal margins, and continued stability in the high-altitude north, together signaling mounting adaptation challenges for water, flood, and disaster management.

#### Late-century (2073–2100)

**Fig. 12:**
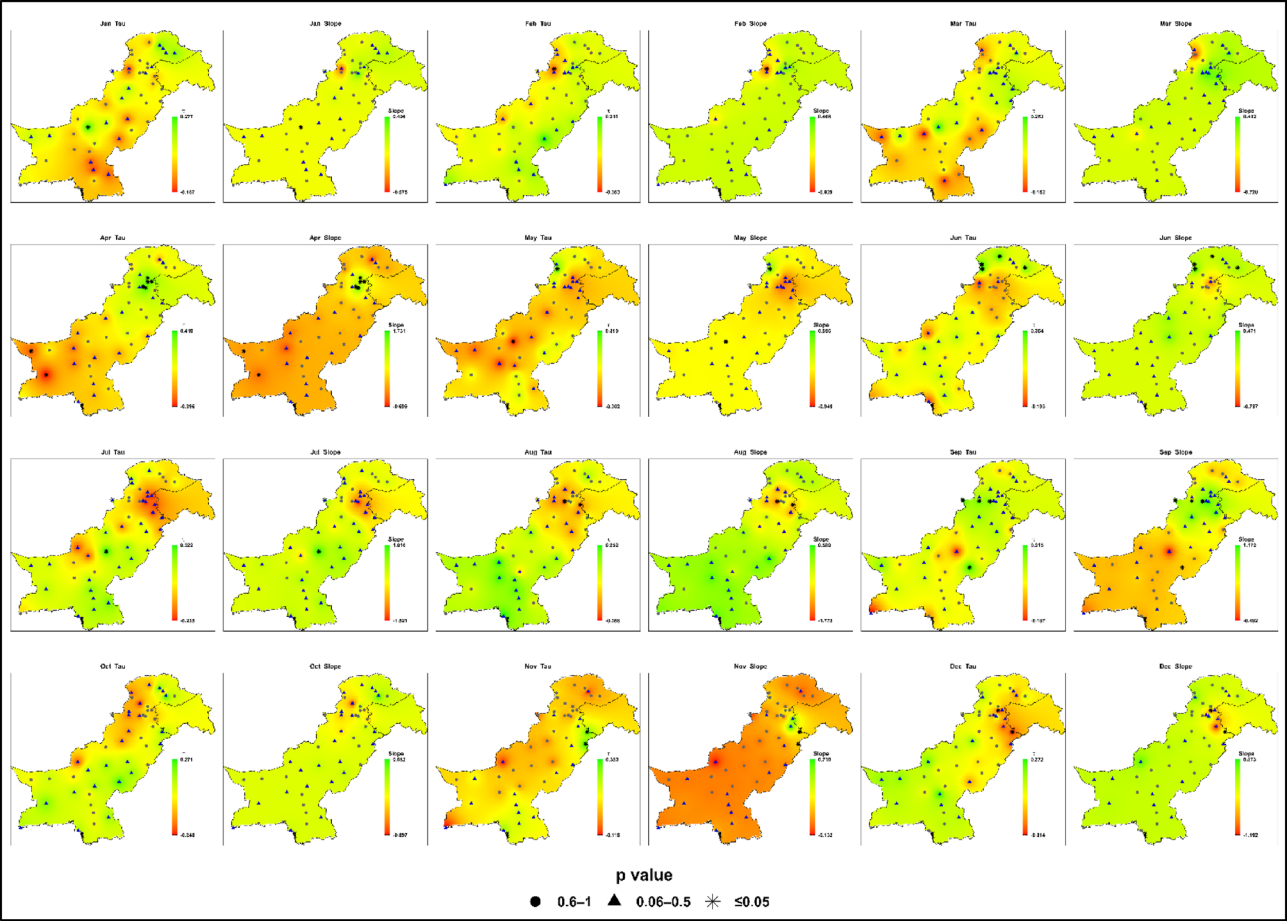
Spatial distributions of Kendall’s Tau and Sen’s slope (mm month⁻¹) for monthly maximum daily precipitation (Rx1day at monthly resolution) across Pakistan under SSP5-8.5 during the late-century period (2073–2100). P-value symbols (★ *p* ≤ 0.05; ▲ 0.06–0.5; ● 0.6–1) represent station-level statistical significance.

Late-century projections under SSP5-8.5 (Fig. 12; Table S7) reveal the strongest intensification and sharpest spatial contrasts in extreme precipitation of all scenarios, with Sen’s slopes ranging from − 2.32 to + 2.16 mm/month. Although most grid-point trends remain statistically insignificant, the magnitude and frequency of extremes exceed those observed during the historical baseline period, although grid-scale trends remain largely statistically insignificant.

Zones 4 and 7 (Potohar, northern Punjab, Kashmir; southern Punjab, upper Sindh) emerge as consistent hotspots, with monsoon maxima routinely reaching 140–150 mm, up to 70% above baseline. The results given in Fig. 5 show that the widest interquartile ranges and most frequent extreme outliers, indicating substantially elevated flood, drainage, and agricultural risks under high-emission conditions for Pakistan’s core population and production centers.

Zone 6 (northwestern highlands) displays highly variable monsoon peaks (up to 125 mm), with both strong positive anomalies and localized declines in monthly maximum daily precipitation, compounding landslide and runoff hazards. Zone 2 (central Punjab, KP) records July-August maxima of 90–100 mm and clear increases in interannual variability. In contrast, Zones 3 and 5 exhibit strengthened negative trends in monthly maximum daily precipitation, with spring and early summer slopes dropping below − 2.0 mm/month, though rare convective outliers (up to 75 mm) persist. Zone 1 (high-altitude north) remains comparatively stable, with maxima of 35–40 mm and only occasional extremes, suggesting continued resilience of glacier-fed hydrology. The results given in Fig. 5 for F3 under SSP5-8.5 display the greatest rainfall variability of all scenarios, with elongated whiskers and a broader spread than historically observed.

## Discussion

These findings confirm the strong emission sensitivity of Pakistan’s hydroclimate. Under high-emission scenarios, monsoon-dominated northeastern and south-central regions are projected to experience the sharpest amplification of extremes, while arid western and coastal belts show persistent weakening of monthly maximum daily precipitation and high-altitude northern zones remain comparatively stable. This spatially heterogeneous but climatically coherent response aligns with broader CMIP6-based assessments of South Asian hydroclimate^49–52^. Importantly, the focus on monthly-scale daily precipitation extremes, rather than seasonal or annual means, highlights short-duration convective bursts that disproportionately trigger floods and landslides^53^. Unlike aggregated approaches, this zonal analysis uncovers hazard-relevant patterns that may otherwise remain underestimated, thereby providing higher precision for risk-sensitive planning^54^.

### Water security and agricultural vulnerability

Late-century SSP5-8.5 projections indicate pronounced monsoon amplification in northeastern and south-central Pakistan, consistent with Rx1day/Rx5day intensification in CMIP6 high-emission ensembles^37^ and linked to Arabian Sea SST warming^55,56^. Similar monsoon escalation has been observed across the eastern Himalaya and Indo-Gangetic Plain, where orographic uplift, low-level jet strengthening, and mesoscale convective systems sustain high-intensity precipitation^57–59^.

These intensifying extremes have important implications for water security and agricultural systems. Khan et al.^60^ showed drought hazards in Swat remain modest under SSP2-4.5 but intensify under SSP5-8.5, while Khan et al.^61^ projected earlier peak flows and destabilized streamflow regimes in the Potohar Plateau. Shah and Chen^62^ highlighted reduced crop yields and rangeland productivity under erratic rainfall and prolonged droughts. Javed et al.^63^ demonstrated that precipitation variability governs crop performance in southwestern Pakistan.

These findings reveal systemic vulnerability: urban centers face rising flood risks^64^, while rural and agricultural systems are increasingly exposed to compound flood-dryness risks documented in the literature^65^. This dual hazard threatens food security and socio-economic stability, underscoring the urgency of adaptive strategies in one of the world’s most climate-vulnerable regions^66^. These water–agriculture linkages directly align with SDG 2 (Zero Hunger) and SDG 6 (Clean Water and Sanitation), as precipitation extremes increasingly determine food security and water availability across Pakistan.

### Emission pathway dependence

The analysis highlights a strong dependence of future precipitation extremes on emission pathways, with SSP2-4.5 yielding moderate late-century increases while SSP5-8.5 produces much larger surges, consistent with regional CMIP6 assessments^67,68^. Smaller increases reported by Tanwar et al.^69^ likely reflect reliance on seasonal aggregation, which tends to obscure short-duration extremes^42^.

Recent studies further emphasize sharp spatial contrasts. Northern highlands exhibit heightened vulnerability, with one-day rainfall extremes projected to intensify under high-emission^30^, while the Kashmir-Jhelum Basin shows destabilized hydroclimatic regimes linked to concurrent warming and changes in precipitation characteristics^70^. Agricultural consequences are similarly heterogeneous scenarios: precipitation variability drives divergent crop outcomes in the southwest^63^, whereas intensifying extremes in central rangelands threaten yields and pastoral systems^62^.

Water scarcity compounds these risks. Independent studies project more frequent severe droughts even under flood-prone conditions^71–73^, alongside intensified but uncertain streamflow regimes in glacier-fed basins, raising concerns for irrigation and hydropower reliability^74^.

The interaction of emission pathways and regional contrasts suggests that Pakistan’s future extremes will not be uniform: volatile monsoon intensification dominates in the northeast and south, persistent drying characterizes the arid west and coast, and highland hydrology remains highly uncertain. This duality of flood risk underscores the need for integrated, zone-specific adaptation strategies. The contrasting futures under SSP2-4.5 and SSP5-8.5 underscore the critical importance of mitigation, directly supporting SDG 13 (Climate Action), where emission trajectories dictate the scale of future hydroclimatic risk.

### Mechanistic drivers of projected changes

The intensification of extremes in Pakistan arises from orographic uplift, thermodynamic moisture loading, and circulation anomalies^49,52^. Orographic forcing amplifies rainfall in windward zones such as Potohar, Hazara, and Kashmir, while adjacent leeward valleys remain comparatively drier, driving sharp intra-zonal contrasts^14,75^. Warming enhances convective bursts in northeastern and southern basins through increased atmospheric moisture availability, while western Balochistan and the southern coast remain predominantly arid, punctuated by episodic cyclonic activity and monsoon-withdrawal disturbances rather than sustained wet conditions^76,77^. Recent studies show that high-elevation and orographically complex regions experience disproportionate increases in short-duration precipitation extremes, even where changes in mean precipitation are limited^30,42^, Kashmir-Jhelum exhibits destabilized hydroclimatic regimes^70^, and Balochistan rainfall remains strongly modulated by westerly disturbance variability^22^. Broader teleconnections, including CO₂ and SST anomalies^78^, internal monsoon energetic modes (Lu et al., 2025), and jet moisture anomalies implicated in the 2010 and 2022 floods^79^, further reinforce rainfall volatility. Collectively, these drivers underscore that Pakistan’s future extremes will be shaped by both thermodynamic forcing and circulation shifts, yielding stronger monsoon-season extreme precipitation in core regions and persistently weaker extreme-event magnitudes in western arid zones. Understanding these thermodynamic and circulation drivers is central to anticipating risks, directly supporting SDG 13 (Climate Action) while also informing SDG 9 (Industry, Innovation, and Infrastructure) through resilient design standards.

### Regional contrasts and inter-zonal variability

Projected extremes reveal sharp inter-zonal contrasts: northeastern (Zone 4) and south-central (Zone 7) basins emerge as consistent hotspots of flood risk^80^, while western Balochistan and the southern coast (Zones 3 and 5) trend toward weaker monthly maximum daily precipitation extremes, punctuated by erratic storms^81,82^. The high-altitude north (Zone 1) remain comparatively stable in extreme rainfall magnitudes but are highly sensitive to small maxima shifts that can accelerate glacier melt and heighten GLOF risk^83^. These contrasts highlight the need for zone-specific adaptation, urban flood and water infrastructure upgrades in the northeast and south, broader hydroclimate resilience, including drought preparedness and groundwater governance in the west and coast, and glacier- and slope-focused risk reduction in the north, within a coherent national framework. Such spatial contrasts emphasize the need for differentiated strategies, directly tied to SDG 11 (Sustainable Cities and Communities) through urban resilience, and SDG 15 (Life on Land) through sustainable land and ecosystem management.

### Limitations

The findings of this study should be interpreted in light of several methodological constraints. The use of CMIP6 global climate models, with typical spatial resolutions of ~ 100 km, limits their ability to capture localized convective systems and mesoscale dynamics that drive extreme precipitation in Pakistan’s complex terrain. This constraint is particularly critical in orographically sensitive regions such as the northwestern highlands, where small-scale processes govern runoff generation and landslide hazards. Model validation is further challenged by the sparse and uneven distribution of ground stations, especially in Balochistan and the northern highlands, reducing confidence in regionalized diagnostics. While ensemble weighting reduces individual model bias and enhances robustness, substantial inter-model spread remains, particularly in transitional climate zones, underscoring the uncertainty of future hydroclimatic projections. Moreover, the analytical focus on monthly-scale daily precipitation extremes, while hazard-relevant, does not directly capture hydrological responses such as streamflow variability, rainfall-snowmelt interactions, or return periods. Addressing these gaps will require integration of high-resolution regional climate models, couple hydro-climatic simulations, and expanded observational networks to improve process representation and spatial fidelity. Closing these knowledge gaps will not only improve scientific accuracy but also strengthen the evidence base for achieving SDG-linked adaptation planning.

### Policy recommendations

This study highlights the urgent need to integrate climate projections into Pakistan’s water, agriculture, and disaster management frameworks. Intensifying monsoon extremes demand investments in urban flood defenses, early-warning systems, and slope stabilization in vulnerable basins, while western and coastal regions require integrated hydroclimate risk management, including drought resilience and groundwater governance. Agricultural policy should encourage diversification and climate-resilient crop varieties to buffer against both excess rainfall and seasonal scarcity. At the same time, research gaps, particularly sparse observational networks and limited high-resolution modeling, must be addressed to better translate rainfall extremes into actionable planning. Given the spatial contrasts identified, adaptation must differentiate between flood-prone monsoon regions and drought-prone arid/coastal regions. Coordinated national policies that embed climate science into infrastructure design, basin-scale water allocation, and food security planning are essential to reduce escalating risks. While drought resilience is discussed here in a policy context, it is informed by the broader literature rather than directly inferred from the Rx1day-month analysis presented in this study. These policy directions directly reinforce SDG 2 (Zero Hunger), SDG 6 (Clean Water and Sanitation), SDG 11 (Sustainable Cities and Communities), and SDG 13 (Climate Action), ensuring that adaptation strategies are aligned with global sustainability goals.

### Conclusion

This study demonstrates that extreme precipitation in Pakistan is strongly emission-dependent, with SSP5-8.5 producing the most pronounced monsoon amplification. By analyzing monthly maximum daily precipitation rather than seasonal means, the results provide hazard-relevant insights that reveal localized vulnerabilities often masked in aggregated assessments. These findings confirm that northeastern and south-central Pakistan face escalating flood risks, while western and coastal regions are more exposed to prolonged aridity and episodic storms, and northern highlands remain comparatively stable but sensitive to even small shifts. Beyond quantifying these spatial contrasts, the study underscores the urgency of embedding climate science into national adaptation policy, ensuring that infrastructure design, agricultural planning, and water governance account for the rising volatility of Pakistan’s hydroclimate. By linking climate science with adaptation planning, this study advances the evidence base needed to achieve SDG 13 (Climate Action), while contributing to water, food, and urban resilience under interconnected SDGs.

## Supplementary Information

Below is the link to the electronic supplementary material.

Supplementary Material 1

## Data Availability

The model data (CMIP6) is available online and can be accessed on the link: https://wcrp-cmip.org/cmip-phases/cmip6/. The observed data will be made available on request.
