# Supplementary material for: Future outlook of monthly maximum daily precipitation in Pakistan’s hydroclimatic zones: high-resolution insights from CMIP6 multimodel data
Source: Sci Rep. 2026 Apr 4;16:16330. doi: 10.1038/s41598-026-45047-6 (PMC13212918; doi:10.1038/s41598-026-45047-6)
Supplement: Supplementary file 1 — Supplementary Material 1 [file 41598_2026_45047_MOESM1_ESM.docx]

Table S1. Baseline (1985–2014) trends in monthly maximum daily precipitation across Pakistan.

**Note:** *For each month, reported p-values indicate the minimum and maximum values across all stations within the study domain, reflecting the range of station-level trend significance, applies to tables (S1–S7).*

| **Month** | **p-value (min)** | **p-value (max)** |
| --- | --- | --- |
| Jan | 0.112 | 0.997 |
| Feb | 0.019 | 0.984 |
| Mar | 0.006 | 0.905 |
| Apr | 0.132 | 0.965- |
| May | 0.05 | 0.999 |
| Jun | 0.011 | 0.997 |
| Jul | 0.032 | 0.998 |
| Aug | 0.011 | 0.999 |
| Sep | 0.025 | 1.000 |
| Oct | 0.04 | 0.984 |
| Nov | 0.036 | 0.985 |
| Dec | 0.01 | 0.985 |

Table S2. Projected near-future (2017–2044) trends in monthly maximum daily precipitation under SSP2-4.5.

| **Month** | **p-value (min)** | **p-value (max)** |
| --- | --- | --- |
| Jan | 0.066 | 0.999 |
| Feb | 0.022 | 0.971 |
| Mar | 0.014 | 0.980 |
| Apr | 0.025 | 0.954 |
| May | 0.032 | 0.984 |
| Jun | 0.004 | 0.997 |
| Jul | 0.008 | 0.987 |
| Aug | 0.007 | 0.999 |
| Sep | 0.016 | 0.970 |
| Oct | 0.067 | 0.968 |
| Nov | 0.023 | 0.980 |
| Dec | 0.053 | 1.000 |

Table S3. Projected mid-century (2045–2072) trends in monthly maximum daily precipitation under SSP2-4.5.

| **Month** | **p-value (min)** | **p-value (max)** |
| --- | --- | --- |
| Jan | 0.087 | 0.997 |
| Feb | 0.020 | 0.988 |
| Mar | 0.007 | 0.984 |
| Apr | 0.021 | 0.988 |
| May | 0.039 | 0.950 |
| Jun | 0.003 | 0.936 |
| Jul | 0.010 | 0.996 |
| Aug | 0.020 | 0.983 |
| Sep | 0.006 | 0.999 |
| Oct | 0.006 | 0.985 |
| Nov | 0.020 | 0.995 |
| Dec | 0.037 | 0.989 |

Table S4. Projected late-century (2073–2100) trends in monthly maximum daily precipitation (Rx1day) under SSP2-4.5.

| **Month** | **p-value (min)** | **p-value (max)** |
| --- | --- | --- |
| Jan | 0.108 | 0.989 |
| Feb | 0.008 | 0.999 |
| Mar | 0.023 | 0.999 |
| Apr | 0.073 | 0.999 |
| May | 0.017 | 0.999 |
| Jun | 0.143 | 1.000 |
| Jul | 0.035 | 0.982 |
| Aug | 0.013 | 0.999 |
| Sep | 0.023 | 1.000 |
| Oct | 0.011 | 0.982 |
| Nov | 0.006 | 0.996 |
| Dec | 0.019 | 0.977 |

Table S5. Projected near-future (2017–2044) trends in monthly maximum daily precipitation (Rx1day) under SSP5-8.5.

| **Month** | **p-value (min)** | **p-value (max)** |
| --- | --- | --- |
| Jan | 0.056 | 0.979 |
| Feb | 0.035 | 0.971 |
| Mar | 0.076 | 0.993 |
| Apr | 0.076 | 1.000 |
| May | 0.018 | 0.990 |
| Jun | 0.011 | 0.926 |
| Jul | 0.049 | 0.998 |
| Aug | 0.001 | 0.997 |
| Sep | 0.043 | 0.999 |
| Oct | 0.066 | 0.998 |
| Nov | 0.012 | 0.971 |
| Dec | 0.035 | 0.970 |

Table S6. Projected mid-century (2045–2072) trends in monthly maximum daily precipitation (Rx1day) under SSP5-8.5.

| **Month** | **p-value (min)** | **p-value (max)** |
| --- | --- | --- |
| Jan | 0.008 | 0.970 |
| Feb | 0.007 | 0.988 |
| Mar | 0.044 | 0.998 |
| Apr | 0.077 | 0.999 |
| May | 0.040 | 1.000 |
| Jun | 0.064 | 0.998 |
| Jul | 0.023 | 0.996 |
| Aug | 0.023 | 0.942 |
| Sep | 0.023 | 0.995 |
| Oct | 0.001 | 0.969 |
| Nov | 0.065 | 0.998 |
| Dec | 0.043 | 0.999 |

Table S7. Projected late-century (2073–2100) trends in monthly maximum daily precipitation (Rx1day) under SSP5-8.5.

| **Month** | **p-value (min)** | **p-value (max)** |
| --- | --- | --- |
| Jan | 0.051 | 0.998 |
| Feb | 0.016 | 1.000 |
| Mar | 0.074 | 0.999 |
| Apr | 0.006 | 0.963 |
| May | 0.027 | 0.978 |
| Jun | 0.013 | 0.964 |
| Jul | 0.023 | 1.000 |
| Aug | 0.026 | 0.996 |
| Sep | 0.027 | 0.999 |
| Oct | 0.060 | 0.999 |
| Nov | 0.012 | 0.998 |
| Dec | 0.041 | 0.998 |
